# Long‐term exposure to increasing temperature can offset predicted losses in marine food quality (fatty acids) caused by ocean warming

**DOI:** 10.1111/eva.13059

**Published:** 2020-07-28

**Authors:** Peng Jin, Gala Gonzàlez, Susana Agustí

**Affiliations:** ^1^ Red Sea Research Center (RSRC) King Abdullah University of Science and Technology (KAUST) Thuwal Saudi Arabia; ^2^ School of Environmental Science and Engineering Guangzhou University Guangzhou China

**Keywords:** fatty acids, food quality, lipids, ocean warming, phenotypic plasticity, physiological adaptation, PUFA

## Abstract

Marine phytoplankton produce essential fatty acids (FA), which are key component of a healthy diet in humans and marine food webs. Increased temperatures can reduce lipid and FA content in phytoplankton; thus, ocean warming poses a risk for the global production of these essential FA. However, responses to warming may differ between phytoplankton species especially after long‐term exposure because phenotypic plasticity, *de novo* mutations, or genetic evolution may occur. Here, we examine the content of FA and lipids in phytoplankton following long‐term selection (~2 years) to warming conditions (+4°C), and we observe that FA and lipids content were partly or entirely recovered following long‐term exposure to warming conditions. Furthermore, this observed long‐term response also offset the predicted losses of some essential polyunsaturated fatty acids (PUFA) in three of the four species tested. Our study suggests that long‐term exposure of phytoplankton to warming may help to maintain marine food quality in a moderately warming ocean. The responses of FA to increasing temperatures may vary among species, and the level of this idiosyncrasy remains to be further studied.

## 1 INTRODUCTION

Phytoplankton account for nearly 50% of the earth’s net primary production and produce many complex biomolecules (Field et al., 1998), including lipids and fatty acids (FA) (Guschina & Harwood, 2009). Lipids and FA are crucial components of cells, for example, they play critical roles in cell membrane function, physiological processes in organisms for energy storage, and trophic interactions in aquatic food webs (Dalsgaard et al., 2003; Guschina & Harwood, 2009).

The lipid contents and FA compositions in phytoplankton are highly dependent on environmental conditions such as CO_2_ concentration (Leu et al., 2013; Torstensson et al., 2013; Wang et al., 2017), temperature (James et al., 1989; Torstensson et al., 2013; Hixson and Arts, 2016), and nutrient availability (Harrison et al., 1990; Reitan et al., 1994; Roleda et al., 2013). FA consist of hydrocarbon chains of different lengths and saturation (number of double bonds), and they are generally classified into three groups: saturated (SFA, no double bonds), monounsaturated (MUFA, one double bond), and polyunsaturated fatty acid (PUFA, with two or more double bonds). Phytoplankton regulate their FA compositions and the degree of desaturation in response to changing temperature to keep a steady membrane fluidity (Guschina & Harwood, 2009). In general, phytoplankton increase the membrane PUFA content in response to decreasing temperatures in order to maintain fluidity, as the double bonds in PUFA increase flexibility and enhance the ability of a FA to "bend," therefore leading to increased membrane fluidity (Thompson et al., 1992; Guschina & Harwood, 2009). In contrast, phytoplankton decrease their membrane PUFA content in response to increasing temperatures while simultaneously increasing SFA, to maintain cell membrane structural rigidity in a less ordered environment (Rousch et al., 2003, Fuschino et al., 2011). PUFA are exclusively synthesized by phytoplankton in aquatic food webs and cannot be synthesized *de novo* by metazoans, and therefore must be acquired by all other organisms via their diet (Hixson et al., 2015). As found by Gladyshev et al., (2013), humans withdraw from aquatic ecosystems about 180 million kg of eicosapentaenoic acid (EPA) and docosahexaenoic acid (DHA) per year. PUFA play a key role in copepod egg production and hatching, zooplankton growth (Jónasdóttir et al., 2005), and fish development (Watanabe et al., 1983) and are important for human health (Riediger et al., 2009). Thus, PUFA composition in phytoplankton is an important determinant of food quality and, consequently, is an important factor in the health and optimal functioning of marine and freshwater food webs (Dalsgaard et al., 2003).

Global change induces many changes in marine environments, such as warming (Gattuso et al., 2015). Hixson & Arts (2016) in a meta‐analysis synthesised nearly 1000 FA profiles of marine and freshwater phytoplankton and found a negative relationship between PUFA and temperature. They predicted that PUFA production in phytoplankton would significantly decrease because of ocean warming. Specifically, global concentrations of n‐3 long‐chain PUFA are predicted to reduce by 8.2% for EPA and 27.8% for DHA with an increase in water temperature of 2.5°C (Hixson & Arts, 2016). However, most studies in the literature have analysed responses in the short term only (i.e., 1–2 weeks) and thus were unable to resolve long‐term responses of phytoplankton to a warming environment. Since phytoplankton have such a large population size, standing genetic variation, and a short generation time (Reusch & Boyd, 2013), they have high potential to adapt to ongoing changes in temperature, as indicated in recent studies (Schaum et al., 2017; Jin & Agustí, 2018).

To investigate the long‐term responses of phytoplankton to ocean warming including through changes in lipid production and FA composition, we conducted a ~2 years experimental evolution experiment using four diatomic microalgae isolated from the Red Sea. The four species of diatoms (*Chaetoceros* sp., *Thalassiosira* sp., *Chaetoceros tenuissimus*, and *Synedra* sp.) were maintained in the laboratory for 2 years at 26°C (ambient temperature control) and at 30°C (experimental warming conditions). 26°C represents the mean Red Sea surface temperature (SST) for the 1982‐2015 period. We selected the projected SST of 30°C (mean Red Sea temperature + 4°C) in accordance with the high‐emission scenario (RCP 8.5, IPCC, 2014) that projects an increase of 2.6–4.8°C by the turn of the next century (2100). We assayed lipid contents and FA composition in the diatoms kept under ambient (26 ± 0.1°C) and warming (30 ± 0.1°C) conditions at the end of ~2‐year selection period. The critical question we wished to answer was whether adaptive evolution to warming leads to complete or partial restoration of phytoplankton lipids and FA content. We also conducted a reciprocal transplant experiment by testing the response of lipids and FA in the long‐term selected strains (ambient and warming strains) to short‐term (1 week) exposure to reciprocal increasing (30°C) and decreasing (26°C) temperatures, respectively.

## 2 METHODS

### 2.1 Culture conditions

Four diatom species *Chaetoceros* sp., *Thalassiosira* sp., *Chaetoceros tenuissimus,* and *Synedra* sp. were isolated from coastal Red Sea waters from Al Fahal Reef (22.2528°N, 38.9612°E). These four diatoms are widely distributed in the warm surface waters of Red Sea, and the diatoms become the predominant phytoplankton in the Red Sea when nutrient availability increases (Kheireddine et al., 2017; Pearman et al., 2016). Ten single clones were isolated for each species and were pooled in one stock culture, which was further incubated at 24°C in a precise temperature‐controlled incubator with a light: dark cycle of 12 hr:12 hr under 50 μmol photons m^−1^ s^−1^. For the experiments, the stock cultures of each diatom species were diluted to separate biological replicate flasks (*n* = 4) and were grown in 200 ml Erlenmeyer flasks at 26 ± 0.1 (experimental ambient, termed as ambient hereafter) and 30 ± 0.1°C (experimental warming, termed as warming hereafter). The warming temperature was selected to agree with the RCP 8.5 scenario for the turn of this century (IPCC, 2014The culture medium was prepared with filtered seawater from the Red Sea taken from the same location of the isolates and enriched with f/4 medium (Guillard & Ryther, 1962). Silicate was added to a concentration of 50 μM. Nitrate and phosphate were added in concentration of 50 μM and 3.125 μM. These cultures grew under 400 μmol photons m^−2^ s^−1^ with a light: dark cycle of 12 hr:12 hr. Four independent replicate semi‐continuous batch cultures (*n* = 4) were run for ~2 years under ambient and warming conditions by renewing the medium every 3 days for *Chaetoceros* sp, *Thalassiosira* sp, and *C. tenuissimus*, and 7 days for *Synedra* sp. due to their lower growth rate. The initial cell concentration was set at 1000 cells/ml, and the medium was partially renewed every 3 or 7 days to restore the cell density to the initial level (i.e., growth batch cycle). The cell densities were maintained within a range of ~5.0 × 10^4^–2.0 × 10^5^ cells/ml for the species of *Chaetoceros* sp, *Thalassiosira* sp, and *Synedra* sp. and ~2.0 × 10^5^–7.0 × 10^5^ for the species *C. tenuissimus* at the time of dilution. By multiplying the number of generations for the cells under ambient or warming selection for each species after 6 months selection as reported in Jin & Agustí (2018) and 4 (the conversion factor from 6 months to 2 years), we could estimate the approximate number of generations for each species under ambient or warming selection at the end of 2 years temperature selection experiment. Here, we show that after the 2 years temperature selection period, the four diatom species of *Chaetoceros* sp., *Thalassiosira* sp., *Chaetoceros tenuissimus,* and *Synedra* sp. had grown approximately for 1756, 1652, 2224, and 753 generations under ambient treatment, respectively. Under warming selection (i.e., 30°C), these four species had grown approximately for 1760, 1572, 2280, and 776 generations, respectively. Although diatoms are able to undergo sexual (oogamous in centric diatoms which produce flagellate gamete) and asexual reproduction, there was no evidence of sexual reproduction in the present study.

The ~2‐year selected strains, both under ambient and warming conditions, were cross‐transferred to the reciprocal ambient or warming temperatures and acclimated for another week (i.e., reciprocal transplant) in a larger volume of 1 L flasks to have more biomass for the following fatty acid analysis. Other culture conditions, such as light intensity, were maintained strictly the same as in the long‐term selection. The initial cell concentration was the same (1000 cells/ml) for the four species. For these reciprocal transplant experiments, three independent cultures were run for each temperature level.

### 2.2 Lipids extraction and determination

At the end of the reciprocal transplant experiments, the cells were harvested by centrifugation (Avanti J‐26 XP Centrifuge Beckman Coulter) (15 min at 7,000 × *g*, 15°C). The wet pellets were then lyophilized (Christ. Alpha 1‐2 LD plus), and the dry cell samples were stored at −80°C until analysis.

The lipids extractions were conducted following the method in ref (Folch et al., 1957) with modifications (Christie 1982). Briefly, ~50 mg of dry biomass was weighed and homogenized in 5 ml chloroform: methanol (2:1, v/v) with 0.01% BHT for 5 min by sonication. The lipid fraction was separated by centrifugation and the total lipid content was calculated gravimetrically once the solvent containing the lipids (chloroform) was completely evaporated under a stream of N_2_. The total lipid extract was subjected to alkaline transesterification (Christie 1982).

### 2.3 Fatty acid composition analysis

After extraction with hexane, the fatty acid methyl esters (FAMEs) were analysed by gas chromatography using an Agilent 5977A GC system equipped with a mass spectrometric (MS) detector and a flame ionization detector (FID), and an HP‐88 column (60 m × 0.25 mm, 0.2 μm). The injection volume was 1 μl with a 10:1 split at an inlet temperature of 250°C. Helium was used as the carrier gas, with a fixed flow of 1 ml/min throughout the temperature program. The initial column temperature was 175°C for 10 min, then was increased to 220°C at 3°C/min and finally maintained at 220°C for 10 min.

The methyl esters were identified by comparing their retention time and mass spectra with those of 37 standard FA mixtures (SIGMA, Supelco Analytical). Quantification of the FAMEs was based on the integration of individual FA peaks in the chromatograms and quantified using a 5‐point calibration curve (25–180 μg/ml) prepared with FAMES standard mixture. The individual FAME concentrations, quantified by GC software (ChemStation B.04.02), were normalized against the internal standard concentration of 100 μg C19‐FAME (nonadecanoic acid 19:0). Hereafter, the total amount of FA methyl esters is referred to as total FA and grouped by affiliation to saturated fatty acids (SFA), monounsaturated fatty acids (MUFA), and polyunsaturated fatty acids (PUFA).

### 2.4 Statistical analysis

A linear mixed effects model (LME) was used to test the interactions between long‐term selection temperature conditions and short‐term assay temperature conditions. For the analysis, the responses (e.g., lipid contents) was considered the dependent variable, selected conditions and assay conditions were the fixed effects, and the replicate was treated as random effect nested within treatment. Model selection entailed fitting a range of models to the data using restricted maximum likelihood (REML) method, starting with the full model with interaction and then a series of reduced models with interaction terms and main effects removed. For multi‐model selection, we computed small sample‐sized corrected Akaike information criterion scores (AIC) and then compared between models by calculating delta AICc values and AICc weights using the “dredge” function in “MuMIn” package (see Table S3 for all tested models’ AICc). LME was fitted to the data using the “nlme” package and was conducted in R (v. 3.6.1). If there is a significant interaction between selection regime and assay conditions, it indicates that selection changes the direction of the reaction norm of the parameters in response to changes in temperature, suggesting a specific long‐term response to warming, but with no reference to fitness. Multiple comparisons of means were performed on significant effects using generalized linear hypothesis test (glht) and Tukey’s test in the package “multcomp”. Principal component analysis (PCA) was used to test the FA composition in the four species by different treatments by restricted estimation maximum likelihood (REML) method. The PCA was performed in JMP software (JMP Pro 14.1.0). Statistical significance was determined using a probability level of α < 0.05.

## 3 RESULTS

### 3.1 Lipid content

**Figure 1:**
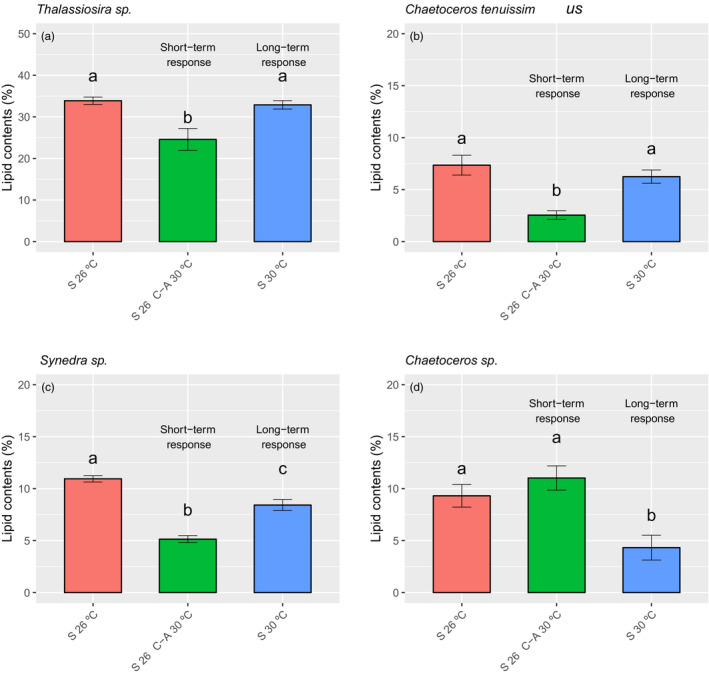
Changes in the content of lipids. The lipid content (% of dry weight) of the different species under different treatments. S 26°C: long‐term (2 years) ambient temperature (26°C) selected cells assayed at 26°C (red bars); S 26°C‐A 30°C: long‐term (2 years) ambient temperature (26°C) selected cells assayed at 30°C (green bars); S 30°C: long‐term (2 years) warming temperature‐selected cells assayed at 30°C (blue bars). (a) *Thalassiosira* sp. (b) *Chaetoceros tenuissimus* (c) *Synedra* sp. and (d) *Chaetoceros* sp. The different letters indicate significant differences between treatments tested by one‐way ANOVA. Values are the average of three replicates and the standard deviation (SD, error bar)

Short‐term warming caused a significant decrease in lipid contents in *Thalassiosira* sp., *C. tenuissimus,* and *Synedra* sp., by 27.4%, 65.3%, and 53.0%, respectively (Figure 1) (Table S1, S2). However, after long‐term selection under warming, the lipid contents were completely or partially restored, with no reduction in long‐term warming‐selected *Thalassiosira* sp. (*p* = .933, Tukey’s test), *C. tenuissimus* (*p* = .157, Tukey’s test) and only a 23% reduction in *Synedra* sp. (*p* < .001, Tukey’s test) (Table S2) (Figure 1) compared with the long‐term ambient selected species. That is, the long‐term warming‐selected populations had higher lipid contents than the ambient selected populations when exposed to the warming temperature (30°C). In contrast, in the species *Chaetoceros sp*., long‐term warming significantly decreased lipids content (% of dry weight) by 53.6% (*p* < .001, Tukey’s test, Table S2) (Figure 1, Table S1), whereas short‐term warming did not show any effect on lipid contents, with no significant differences between ambient and ambient‐exposed to warming treated cells (*p* = .323, Tukey’s test, Table S1) (Figure 1). As compared with growth data, lipid contents in the species of *Thalassiosira* sp. and *Chaetoceros* sp. showed potential trade‐offs with growth rates in the short‐term responses to warming (i.e., the growth significantly increased) (Figure S1). However, they showed similar response patterns with that of growth to long‐term warming in the species of *Thalassiosira* sp. and *Synedra* sp (Figure S1). We observed no correlated responses in the species *Chaetoceros* sp., where there were no significant differences between ambient and warming‐ambient treated cells (*p* = .483, Tukey’s test, Table S2) (Figure 1, Table S1). The significant interactions between selection regime and assay conditions in lipid contents in *Chaetoceros* sp. (LME, Table S3) indicate that selection changes the direction of the reaction norm of lipid contents in response to changes in temperature, suggesting a specific long‐term response to warming.

### 3.2 Fatty acids composition

**Figure 2:**
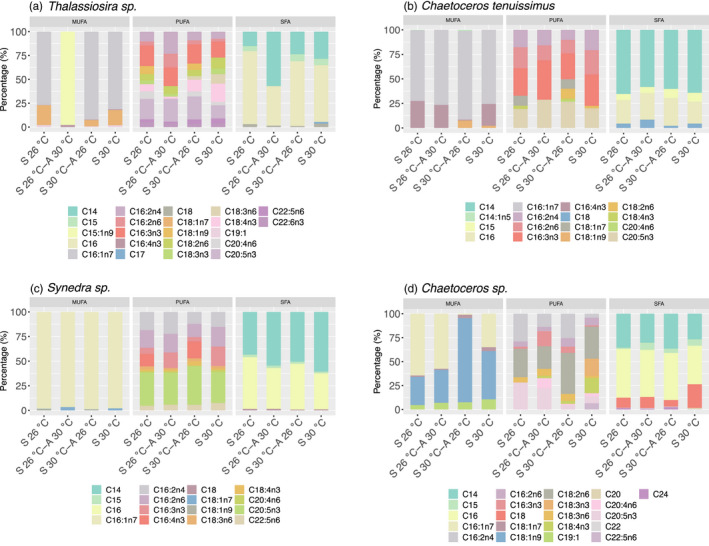
Changes in fatty acid profiles. The percentage (%) of each fatty acid in the four species, (a) *Thalassiosira* sp., (b) *Chaetoceros tenuissimus*, (c) *Synedra* sp., and (d) *Chaetoceros* sp. under the different treatments. S 26^o^C: long‐term (2 years) ambient temperature (26°C) selected cells assayed at 26°C; S 26°C‐A 30°C: long‐term (2 years) ambient temperature (26°C) selected cells assayed at 30°C; S 30°C‐A 26°C: long‐term (2 years) warming temperature (30°C) selected cells assayed at 26°C; S 30°C: long‐term (2 years) warming temperature (30°C) selected cells assayed at 30°C. MUFA: Monounsaturated fatty acid, PUFA: polyunsaturated fatty acid, SFA: Saturated fatty acid. Colors correspond to the different fatty acids abbreviated in the legend. Description of the fatty acids abbreviations is indicated in Table S4

**Figure 3:**
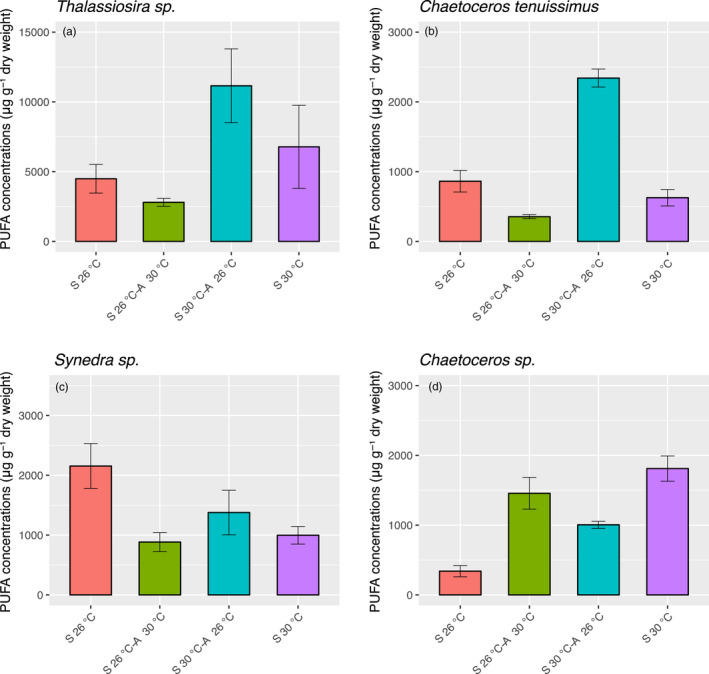
Selection and temperature influence on polyunsaturated fatty acids (PUFA) content. PUFA content (μg/g dry weight) for the different species under different treatments. S 26°C: long‐term (2 years) ambient temperature (26°C) selected cells assayed at 26°C; S 26°C‐A 30°C: long‐term (2 years) ambient temperature (26°C) selected cells assayed at 30°C; S 30°C‐A 26°C: long‐term (2 years) warming temperature (30°C) selected cells assayed at 26°C; S 30°C: long‐term (2 years) warming temperature (30°C) selected cells assayed at 30°C. (a) *Thalassiosira* sp. (b) *Chaetoceros tenuissimus* (c) *Synedra* sp. and (d)* Chaetoceros* sp. Values are the average of three replicates and the standard deviation (SD, error bar)

**Figure 4:**
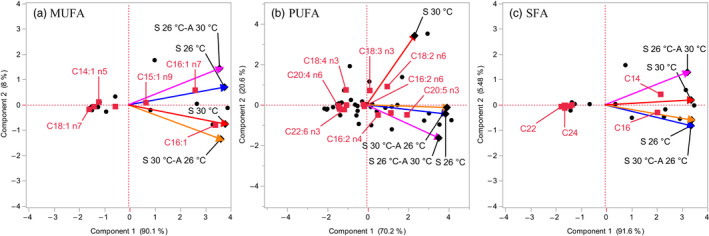
Principal component analysis (PCA) of the composition of FA for the four species tested at the different temperature assays and selective treatments. Monounsaturated (MUFA) (a), polyunsaturated (PUFA) (b), and saturated fatty acid (SFA) (c). S 26°C: long‐term (2 years) ambient temperature (26°C) selected cells assayed at 26°C; S 26°C‐A 30°C: long‐term (2 years) ambient temperature (26°C) selected cells assayed at 30°C; S 30°C‐A 26°C: long‐term (2 years) warming temperature (30°C) selected cells assayed at 26°C; S 30°C: long‐term (2 years) warming temperature (30°C) selected cells assayed at 30°C. The data of all the four species were pooled. Description of the fatty acids abbreviations is indicated in Table S4

A total of 15–20 individual FA were identified and measured in the diatoms *Thalassiosira* sp. and *Chaetoceros* sp. (Figure 2, Table S4). A lower variety of FA compounds was observed in *Synedra* sp. (14–16) and *C. tenuissimus* (10–16) (Figure 2, Table S4). SFA dominated the FA content in all the species, followed by MUFA and PUFA (Figure 2, Table S4). *Thalassiosira* sp. was the species that showed higher FA content (Figure 3) and also synthesized the three essential PUFA, that is, eicosapentaenoic acid (EPA, C20:5n3), docosahexaenoic acid (DHA, C22:6n3), and gamma‐linoleic acid (HTA, C16:3n3) (Figure 2, Table S4). Principal component analysis (PCA) indicated that there were no notable changes in MUFA and SFA composition among the four treatments (correlations 0.75–0.97) (Figure 4). However, we found a substantial change in PUFA composition in long‐term warming‐selected cells with respect to the other three treatments (correlations 0.21–0.48) (Figure 4), suggesting that long‐term warming was sufficient to alter the PUFA composition of the diatoms tested.

#### 3.2.1 Fatty acid composition in *Thalassiosira* sp

The contents of the essential FA of eicosapentaenoic acid (EPA, C20:5n3), docosahexaenoic acid (DHA, C22:6n3), and gamma‐linoleic acid (HTA, C16:3n3) in *Thalassiosira* sp. were resilient both to short‐term and long‐term warming, as no significant differences were observed either between ambient–ambient and ambient–warming treated cells or between ambient–ambient and warming–warming treated cells (Figure 2) (Table S2). However, we found significant interactions between selection temperature and assay temperature on EPA, DHA, and HTA, indicating a specific long‐term response to warming (Table S3). For the PUFA content, neither short‐term (*p* = .714, Tukey’s test) nor long‐term warming (*p* = .480, Tukey’s test) showed a significant effect (Figure 3a). There was however a significant increase in PUFA contents when crossing the temperatures in which the long‐term warming (30°C) selected cells showed significantly higher PUFA contents than long‐term ambient (26°C) selected cells when they were both briefly exposed to short‐term ambient temperature (26°C) (*p* < .001, Tukey’s test) (Figure 3a).

#### 3.2.2 Fatty acid composition in *Chaetoceros tenuissimus*

While short‐term warming decreased the contents of HTA and EPA contents by 41% and 39%, respectively, in *C. tenuissimus*, their contents were partially restored after long‐term warming selection, indicating a specific long‐term response to warming (interaction selection × assay temperature, LME, Table S3). Similarly, total PUFA content in *C. tenuissimus* decreased by 59%, (*p* < .001, Tukey’s test) under short‐term warming, but were completely restored after long‐term warming selection (*p* = .157, Tukey’s test), suggesting an adaptation to warming (Figure 3b). As observed for *Thalassiosira* sp., there was also a significant increase in PUFA contents (*p* < .001, Tukey’s test) when crossing the temperatures in *C. tenuissimus* (Figure 3b).

#### 3.2.3 Fatty acid composition in *Synedra* sp

While short‐term warming did not show any effect on the HTA contents in *Synedra* sp. (*p* = .992, Tukey’s test), long‐term warming positively increased the HTA contents by 47% (*p* = .008, Tukey’s test) (Figure 2, Table S2 and S4). Short‐term warming decreased the EPA contents by 61% (*p* < .001, Tukey’s test); however, this effect was dampened by long‐term warming selection with a 57% reduction in the warming‐selected cells assayed at warming temperature (*p* < .001, Tukey’s test) (Figure 2, Table S2 and S4). We also determined significant interactions between selection temperature and short‐term assay temperature on the contents of HTA and EPA (LME, Table S3). Contrary to that observed for *Thalassiosira* sp. and *C. tenuissimus*, short‐term and long‐term warming negatively decreased the total PUFA contents (both *p* < .001, Tukey’s test) (Figure 3c). The PUFA content was restored, however, when the long‐term warming‐selected cells were shortly exposed to 26°C, with no significant differences between long‐term ambient and warming‐selected cells at 26°C (*p* = .001, Tukey’s test) (Figure 3c).

#### 3.2.4 Fatty acid composition in *Chaetoceros* sp

PUFA in *Chaetoceros sp*. showed a tendency to increase consistently with temperature. Short‐term warming increased the essential FA linoleic acid (LNA, C18:2n6) by 241%. This positive effect was amplified fivefold by long‐term warming (Figure 2, Table S4). There was a positive response of EPA to the short‐term warming selection, where ambient selected populations assayed at warming conditions showed significantly higher EPA contents (239%) than that assayed at ambient conditions (*p* < .001, Tukey’s test). However, this positive effect was muted by long‐term warming, where there was no significant difference between ambient selected‐ambient assayed cells and warming‐selected‐warming assayed cells (*p* = .586, Tukey’s test, Figure 2, Table S4). A positive effect of temperature was also reflected in the total PUFA content, where the short‐term warming increased the content by 329% (*p* < .001, Tukey’s test), and this effect was amplified by long‐term warming with an increase of 434% in the warming‐selected cells when assayed at warming temperature (*p* < .001, Tukey’s test) (Figure 3d). These specific responses of LNA, EPA, and PUFA contents to long‐term warming were further confirmed by the LME analysis (Table S3). When crossing the temperature to 26°C, the long‐term warming‐selected strain of *Chaetoceros* sp. showed reduced PUFA content, but values significantly increased relative to the long‐term ambient selected strain at the same temperature of 26°C (*p* < .001, Tukey’s test) (Figure 3d).

#### 3.2.5 Variation in proportion of FAs between temperature‐selected species

**Figure 5:**
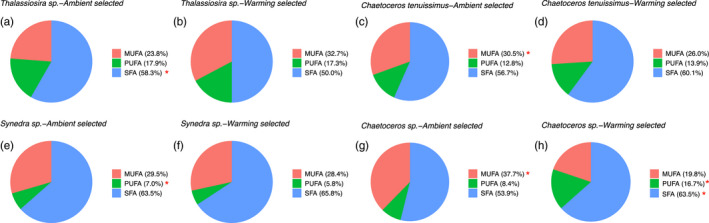
Variation in the proportion of fatty acids saturation between temperature‐evolved species. The pie plots represent the averaged proportion of monounsaturated (MUFA), polyunsaturated (PUFA), and saturated fatty acid (SFA), for the ambient temperature (26°C) and warming temperature (30°C) selected *Thalassiosira* sp. (a, b), *Chaetoceros tenuissimus* (c, d), *Synedra* sp. (e, f) *Chaetoceros* sp. (g, h) The red asterisks indicate the significant differences between ambient‐ and warming‐selected strains

The ability to produce FA after long‐term exposure to temperature was summarized averaging the FA content of the two treatments (i.e., ambient selected and warming selected) for each species. Ambient selected *Thalassiosira* sp. cells showed significantly higher proportions of SFA (*p* = .033) when compared with that of warming‐selected cells, but they did not differ either in MUFA or PUFA proportions (MUFA: *p* = .114; PUFA: *p* = .859) (Figure 5a,b). For the species of *C. tenuissimus*, ambient selected cells showed significantly higher MUFA proportions than warming‐selected cells (*p* < .001), but did not differ in the other two groups (Figure 5c,d). The ambient and warming‐selected *Synedra* sp. cells showed similar SFA and MUFA compositions, but the long‐term warming slightly decreased the PUFA proportions by 17% (*p* = .016) (Figure 5e,f). In contrast to the results observed in the other three species, the FA group of PUFA showed a positive response to the warming selection, where the PUFA proportions almost doubled in the warming‐selected *Chaetoceros* sp. cells (Figure 5g,h).

## 4 DISCUSSION

This study shows how the composition of FA changes of phytoplankton as part of the evolutionary responses to warming in the long term (~2 years) under warming conditions. Here, we show that although the lipids content decreased in three out four diatoms under short‐term warming, the lipids content was partly or entirely restored after long‐term warming exposure (in a warming scenario of 4°C increase). We also observed this restoration in some of the essential FA (e.g., HTA and EPA) and PUFA contents in three out four species tested (e.g., *C. tenuissimus* and *Thalassiosira* sp.). Our study suggests that future marine food quality in a warming ocean will partly depend on the long‐term responses of phytoplankton to their changing environment. These responses may help to counteract the expected decline in marine food quality.

There is growing evidence for adaptation through evolutionary responses of phytoplankton to drivers of global change, such as elevated CO_2_ (Lohbeck et al., 2012; Jin et al., 2013), pollutants (Stachowski‐Haberkorn et al., 2013), and temperature (Schaum et al., 2017; Jin & Agustí 2018). Jin & Agustí demonstrated that the four diatoms tested in the present study showed fast adaptation to warming of 4°C by applying different strategies (Jin & Agustí, 2018). *Chaetoceros* sp. and *Thalassiosira* sp. followed a pattern of changing from “specialist” to “generalist” by shifting the critical thermal minimum and maximum in warming‐selected cells, with no shifts in the optimal growth temperature. However, *C. tenuissimus* and *Synedra* sp. utilized a “hotter is better” strategy to adapt to warming by shifting their optimal growth temperatures and increasing their maximum growth rates under warming conditions (Jin & Agustí, 2018). Lohbeck et al. (2012) demonstrated that calcification rates of the coccolithophore *Emiliania huxleyi* were partly restored in the cells that had selected under increased CO_2_ for 500 generations. Similarly, we observed that lipids production was partly or completely restored in three diatoms (*Thalassiosira* sp., *C. tenuissimus,* and *Synedra* sp.) after long‐term warming selection. The changes in lipids and FA indicate phenotypic buffering, which is by definition adaptive when it confers the maintenance of organismal functioning (Reusch & Boyd, 2013). One possible underlying mechanism for phenotypic buffering is that the genes that are responsible for lipids metabolism were up‐regulated (e.g., malic enzyme, Xue et al., 2015; FA elongases, Cook & Hildebrand, 2016) or silenced (e.g., nitrate reductase, Levitan et al., 2015; pyruvate dehydrogenase kinase, Ma et al., 2014) under warming conditions. The other possible explanation is that in order to maintain the essential contents of lipids, warming‐selected cells acquire additional energy to continue performing lipid metabolism or relocation energy among competing functions (an allocation trade‐off, Angilletta, 2009). In conclusion, the diatom strains in this study responded in a plastic way to accommodate warming and maintain lipid metabolism. However, the reasons accounting for the differences observed between strains/species remains elusive.

Previous studies have shown that PUFA content in phytoplankton is influenced by temperature, describing a negative linear relationship with increasing temperature in a meta‐analysis (Hixson & Arts, 2016). In that study, this environmental influence on the low lipids and PUFA content of tropical species was observed, as the values were lower than those reported for temperate and polar species, with the exception of *Thalassiosira* sp. (Hixson & Arts, 2016). Diatomic microalgae are understood to provide most of the world’s supply of omega‐3 (240 Mt of EPA annually) (Budge et al., 2014), and it is predicted there will be a substantial loss in the future warming ocean (Hixson & Arts, 2016). Since phytoplankton lipids and FA are crucial for various of marine organisms and human health (Ahlgren et al., 2015; Anthony et al., 2009; Larsen et al., 2011; Rossoll et al., 2012; Towle et al., 2015), thus, any changes in phytoplankton lipids and FA content and composition may strongly affect marine food webs and marine‐based diet quality. For instance, one study found that changes in essential FA ratios in the Baltic Sea were transferred to the food web, and lead to a chronic reproductive disease in Atlantic salmon (Ahlgren et al., 2015). Long‐chain n‐3 polyunsaturated fatty acids (PUFA), in particular EPA and DHA, have pleiotropic effects and influence the in vivo production of inflammatory components, blood rheology, and membrane functionality (Riediger et al., 2009). Our findings that levels of essential FA may be able to recover under a warming scenario of 4°C in the long term are important, as this recovery ability may mitigate the consequences for seafood quality in the context of global change, thus buffering the negative effects on fishing industries and human health. It is intriguing that the C:N:P of phytoplankton, which is another crucial component of marine food quality, showed similar response pattern between short‐ and long‐term warming (Yvon‐Durocher et al., 2017; Schaum et al., 2018; Schulhof et al., 2019). As global change induces many changes in marine environments, such as ocean acidification, deoxygenation, and decrease in nutrient availability in the upper open ocean (Gattuso et al., 2015), these environmental drivers would also alter the changes documented in the present study.

Our observations suggest that phytoplankton under warming conditions has the potential to offset losses of marine food quality, by helping to reduce losses in lipids content and through recovery of some essential FA that are lost due to exposure to warmer temperatures. However, whether these responses were driven by phenotypic plasticity, *de novo* mutations, or genetic evolution cannot be concluded with the methodology used and the lack of genetic analysis. Our results also show variability between strains or species. Thus, long‐term responses in a larger number of strains/species or species including also other phytoplankton functional groups than diatoms should be tested. Our experiments were carried out on laboratory unispecific cultures where growth conditions were optimized, without competitors or predators. Similar conditions for optimal phytoplankton growth are not sustained in nature for such long periods, thus implying uncertainties for successful long‐term adaptation in the ocean. In summary, our results outline important phytoplankton long‐term responses helping to reduce losses of lipid and FA with increasing warming and can consequently mitigate the consequences of warming on the marine food diet.

## CONFLICT OF INTERESTS

The authors declare that they have no competing interests.

## AUTHOR CONTRIBUTION

P.J. and S.A. conceived and designed the study. P.J. and G.G performed the experiments. All authors analysed and interpreted the data. P.J. and S.A. wrote the manuscript, and all authors discussed and edited the manuscript.

## Supporting information

Supplementary Material

## Data Availability

All data used to evaluate the conclusions in the paper will be archived at the Dryad Digital Repository upon the acceptance of this manuscript.
