# Supplementary material for: Long‐term exposure to increasing temperature can offset predicted losses in marine food quality (fatty acids) caused by ocean warming
Source: Evol Appl. 2020 Jul 28;13(9):2497–506. doi: 10.1111/eva.13059 (PMC7513733; doi:10.1111/eva.13059)
Supplement: Supplementary file 1 — Supplementary Material [file EVA-13-2497-s001.docx]

**Fig S1.** Changes in the growth rates. The growth rates (d^-1^) of different species under different treatments. S 26 ^o^C: long-term (2 years) ambient temperature (26 ^o^C) selected cells assayed at ambient temperature (26 ^o^C) (red bars); S 26 ^o^C-A 30 ^o^C: long-term (2 years) ambient temperature (26 ^o^C) selected cells assayed at 30 ^o^C (green bars); S 30 ^o^C: long-term (2 years) warming temperature (30 ^o^C) selected cells assayed at warming temperature (30 ^o^C) (blue bars). (a) *Thalassiosira* sp*.* (b) *Chaetoceros tenuissimus* (c) *Synedra* sp. and (d) *Chaetoceros* sp*.* The different letters indicate significant differences between treatments tested by post hoc Tukey’s test. Values are the average of three replicates and the standard deviation (SD, error bar).

**Table S1.** The lipid contents (% of dry weight) of the different species under different treatments. S 26 ^o^C: long-term (2 years) ambient temperature (26 ^o^C) selected cells assayed at 26 ^o^C; S 26 ^o^C-A 30 ^o^C: long-term (2 years) ambient temperature (26 ^o^C) selected cells assayed at 30 ^o^C; S 30 ^o^C-A 26 ^o^C: long-term (2 years) warming temperature (30 ^o^C) selected cells assayed at 26 ^o^C; S 30 ^o^C: long-term (2 years) warming temperature (30 ^o^C) selected cells assayed at 30 ^o^C. Values are the average of three replicates and the standard deviation (SD, in parentheses).

| Species | Treatment | | | |
| --- | --- | --- | --- | --- |
|  | S 26 ^o^C | S 26 ^o^C-A 30 ^o^C | S 30 ^o^C-A 26 ^o^C | S 30 ^o^C |
| *Thalassiosira* sp. | 33.7 (0.8) | 24.6 (2.62) | 29.3 (1.74) | 32.9 (1.01) |
| *Chaetoceros tenuissimus* | 7.35 (0.96) | 2.55 (0.42) | 4.81 (0.43) | 6.25 (0.64) |
| *Synedra* sp. | 10.9 (0.31) | 5.15 (0.34) | 10.1 (0.40) | 8.42 (0.53) |
| *Chaetoceros* sp. | 9.31 (1.10) | 11.0 (1.17) | 7.87 (1.47) | 4.32 (1.20) |

**Table S2.** Statistical analysis of the contents of lipid, eicosapentaenoic acid (EPA, 20:5 n3), docosahexaenoic acid (DHA, 22:6 n3), gamma-linoleic acid (HTA, C16:3n3), linoleic acid (LNA, C18:2n6) and polyunsaturated fatty acids (PUFA) to assess the short-term, long-term, direct and correlated response to warming. Multiple comparisons of means were performed on significant effects using generalized linear hypothesis test (glht) and Tukey’s test. Statistical significance was determined using a probability level of α<0.05. Short-term warming: S 26 ^o^C vs S 26 ^o^C-A 30 ^o^C; long-term warming: S 26 ^o^C vs S 30 ^o^C; Direct response: S 26 ^o^C-A 30 ^o^C vs S 30 ^o^C; Correlated response: S 26 ^o^C vs S 30 ^o^C-A 26 ^o^C.

| Species | Trait | Response | *p*-value |
| --- | --- | --- | --- |
| *Chaetoceros* sp*.* | Lipid | Short-term warming | *p* = 0.323 |
|  |  | Long-term warming | *p* < 0.001 |
|  |  | Direct response | *p* < 0.001 |
|  |  | Correlated response | *p* = 0.483 |
|  |  |  |  |
|  | EPA | Short-term warming | *p* < 0.001 |
|  |  | Long-term warming | *p* = 0.586 |
|  |  | Direct response | *p* < 0.001 |
|  |  | Correlated response | *p* = 0.035 |
|  |  |  |  |
|  | LNA | Short-term warming | *p* < 0.001 |
|  |  | Long-term warming | *p* = 0.959 |
|  |  | Direct response | *p* < 0.001 |
|  |  | Correlated response | *p* = 0.547 |
|  |  |  |  |
|  | PUFA | Short-term warming | *p* < 0.001 |
|  |  | Long-term warming | *p* < 0.001 |
|  |  | Direct response | *p* = 0.008 |
|  |  | Correlated response | *p* < 0.001 |
|  |  |  |  |
| *Thalassiosira* sp. | Lipid | Short-term warming | *p* < 0.001 |
|  |  | Long-term warming | *p* = 0.933 |
|  |  | Direct response | *p* < 0.001 |
|  |  | Correlated response | *p* = 0.017 |
|  |  |  |  |
|  | EPA | Short-term warming | *p* = 0.888 |
|  |  | Long-term warming | *p* = 1.000 |
|  |  | Direct response | *p* = 0.916 |
|  |  | Correlated response | *p* < 0.001 |
|  |  |  |  |
|  | DHA | Short-term warming | *p* = 0.506 |
|  |  | Long-term warming | *p* = 0.969 |
|  |  | Direct response | *p* = 0.252 |
|  |  | Correlated response | *p* = 0.379 |
|  |  |  |  |
|  | HTA | Short-term warming | *p* = 0.531 |
|  |  | Long-term warming | *p* = 0.951 |
|  |  | Direct response | *p* = 0.236 |
|  |  | Correlated response | *p* < 0.001 |
|  |  |  |  |
|  | PUFA | Short-term warming | *p* = 0.714 |
|  |  | Long-term warming | *p* = 0.480 |
|  |  | Direct response | *p* = 0.061 |
|  |  | Correlated response | *p* < 0.001 |
|  |  |  |  |
| *Chaetoceros tenuissimus* | Lipid | Short-term warming | *p* < 0.001 |
|  |  | Long-term warming | *p* = 0.157 |
|  |  | Direct response | *p* < 0.001 |
|  |  | Correlated response | *p* < 0.001 |
|  |  |  |  |
|  | EPA | Short-term warming | *p* < 0.001 |
|  |  | Long-term warming | *p* = 0.048 |
|  |  | Direct response | *p* = 0.497 |
|  |  | Correlated response | *p* < 0.001 |
|  |  |  |  |
|  | HTA | Short-term warming | *p* = 0.070 |
|  |  | Long-term warming | *p* = 0.741 |
|  |  | Direct response | *p* = 0.484 |
|  |  | Correlated response | *p* < 0.001 |
|  |  |  |  |
|  | PUFA | Short-term warming | *p* < 0.001 |
|  |  | Long-term warming | *p* = 0.066 |
|  |  | Direct response | *p* = 0.024 |
|  |  | Correlated response | *p* < 0.001 |
|  |  |  |  |
| *Synedra* sp*.* | Lipid | Short-term warming | *p* < 0.001 |
|  |  | Long-term warming | *p* < 0.001 |
|  |  | Direct response | *p* < 0.001 |
|  |  | Correlated response | *p* = 0.040 |
|  |  |  |  |
|  | EPA | Short-term warming | *p* < 0.001 |
|  |  | Long-term warming | *p* < 0.001 |
|  |  | Direct response | *p* = 0.993 |
|  |  | Correlated response | *p* = 0.174 |
|  |  |  |  |
|  | HTA | Short-term warming | *p* = 0.992 |
|  |  | Long-term warming | *p* = 0.008 |
|  |  | Direct response | *p* = 0.019 |
|  |  | Correlated response | *p* < 0.001 |
|  |  |  |  |
|  | PUFA | Short-term warming | *p* < 0.001 |
|  |  | Long-term warming | *p* < 0.001 |
|  |  | Direct response | *p* = 0.948 |
|  |  | Correlated response | *p* = 0.001 |

**Table S3.** The linear mixed effects models of the interactions between selective temperature conditions and assay temperature conditions on contents of lipid, eicosapentaenoic acid (EPA, 20:5 n3), docosahexaenoic acid (DHA, 22:6 n3), gamma-linoleic acid (HTA, C16:3n3), linoleic acid (LNA, C18:2n6) and polyunsaturated fatty acids (PUFA). For the analysis, the responses (e.g. lipid contents) was considered the dependent variable, selected conditions and assay conditions were the fixed effects, and the replicate was treated as random effect nested within treatment. The most parsimonious model is highlighted in bold.

| Species | Trait | Model structure | df | AICc |
| --- | --- | --- | --- | --- |
| *Chaetoceros* sp*.* | Lipid contents | **Lipids ~ selection * assay** | 6 | 22.9 |
|  |  | Lipids ~ selection + assay | 5 | 27.5 |
|  |  | Lipids ~ selection | 4 | 39.2 |
|  |  | Lipids ~ assay | 4 | 29.8 |
|  |  | Lipids ~ 1 | 3 | 39.8 |
|  | EPA contents | **EPA ~ selection * assay** | 6 | 111.3 |
|  |  | EPA ~ selection + assay | 5 | 129.7 |
|  |  | EPA ~ selection | 4 | 136.6 |
|  |  | EPA ~ assay | 4 | 134.7 |
|  |  | EPA~ 1 | 3 | 143.0 |
|  | LNA contents | **HTA ~ selection * assay** | 6 | 116.0 |
|  |  | HTA ~ selection + assay | 5 | 129.7 |
|  |  | HTA ~ selection | 4 | 135.6 |
|  |  | HTA ~ assay | 4 | 136.7 |
|  |  | HTA~ 1 | 3 | 143.8 |
|  | PUFA contents | **PUFA ~ selection * assay** | 6 | 135.9 |
|  |  | PUFA ~ selection + assay | 5 | 142.5 |
|  |  | PUFA ~ selection | 4 | 171.9 |
|  |  | PUFA ~ assay | 4 | 161.2 |
|  |  | PUFA~ 1 | 3 | 183.0 |
|  |  |  |  |  |
| *Thalassiosira* sp. | Lipid contents | Lipids ~ selection * assay | 6 | 8.6 |
|  |  | **Lipids ~ selection + assay** | 5 | 7.9 |
|  |  | Lipids ~ selection | 4 | 19.3 |
|  |  | Lipids ~ assay | 4 | 18.9 |
|  |  | Lipids ~ 1 | 3 | 28.5 |
|  | EPA contents | **EPA ~ selection * assay** | 6 | 155.0 |
|  |  | EPA ~ selection + assay | 5 | 166.1 |
|  |  | EPA ~ selection | 4 | 179.7 |
|  |  | EPA ~ assay | 4 | 179.4 |
|  |  | EPA~ 1 | 3 | 193.1 |
|  | DHA contents | **DHA ~ selection * assay** | 6 | 130.5 |
|  |  | DHA ~ selection + assay | 5 | 133.1 |
|  |  | DHA ~ selection | 4 | 140.0 |
|  |  | DHA ~ assay | 4 | 142.2 |
|  |  | DHA~ 1 | 3 | 150.1 |
|  | HTA contents | **HTA ~ selection * assay** | 6 | 150.9 |
|  |  | HTA ~ selection + assay | 5 | 158.5 |
|  |  | HTA ~ selection | 4 | 172.7 |
|  |  | HTA ~ assay | 4 | 175.0 |
|  |  | HTA~ 1 | 3 | 187.8 |
|  | PUFA contents | **PUFA ~ selection * assay** | 6 | 177.9 |
|  |  | PUFA ~ selection + assay | 5 | 187.8 |
|  |  | PUFA ~ selection | 4 | 202.8 |
|  |  | PUFA ~ assay | 4 | 209.0 |
|  |  | PUFA~ 1 | 3 | 223.5 |
|  |  |  |  |  |
| *Chaetoceros tenuissimus* | Lipid contents | Lipids ~ selection * assay | 6 | 33.3 |
|  |  | **Lipids ~ selection + assay** | 5 | 27.6 |
|  |  | Lipids ~ selection | 4 | 37.4 |
|  |  | Lipids ~ assay | 4 | 40.3 |
|  |  | Lipids ~ 1 | 3 | 48.8 |
|  | EPA contents | **EPA ~ selection * assay** | 6 | 103.6 |
|  |  | EPA ~ selection + assay | 5 | 137.4 |
|  |  | EPA ~ selection | 4 | 151.2 |
|  |  | EPA ~ assay | 4 | 149.3 |
|  |  | EPA~ 1 | 3 | 161.8 |
|  | HTA contents | **HTA ~ selection * assay** | 6 | 118.5 |
|  |  | HTA ~ selection + assay | 5 | 133.6 |
|  |  | HTA ~ selection | 4 | 146.3 |
|  |  | HTA ~ assay | 4 | 148.6 |
|  |  | HTA~ 1 | 3 | 159.1 |
|  | PUFA contents | **PUFA ~ selection * assay** | 6 | 132.1 |
|  |  | PUFA ~ selection + assay | 5 | 156.4 |
|  |  | PUFA ~ selection | 4 | 176.7 |
|  |  | PUFA ~ assay | 4 | 173.3 |
|  |  | PUFA~ 1 | 3 | 190.0 |
|  |  |  |  |  |
| *Synedra* sp*.* | Lipid contents | Lipids ~ selection * assay | 6 | 32.2 |
|  |  | **Lipids ~ selection + assay** | 5 | 28.5 |
|  |  | Lipids ~ selection | 4 | 29.2 |
|  |  | Lipids ~ assay | 4 | 40.2 |
|  |  | Lipids ~ 1 | 3 | 39.9 |
|  | EPA contents | **EPA ~ selection * assay** | 6 | 131.4 |
|  |  | EPA ~ selection + assay | 5 | 136.8 |
|  |  | EPA ~ selection | 4 | 153.3 |
|  |  | EPA ~ assay | 4 | 142.1 |
|  |  | EPA~ 1 | 3 | 160.5 |
|  | HTA contents | **HTA ~ selection * assay** | 6 | 106.8 |
|  |  | HTA ~ selection + assay | 5 | 118.4 |
|  |  | HTA ~ selection | 4 | 126.6 |
|  |  | HTA ~ assay | 4 | 120.5 |
|  |  | HTA~ 1 | 3 | 130.7 |
|  | PUFA contents | **PUFA ~ selection * assay** | 6 | 146.0 |
|  |  | PUFA ~ selection + assay | 5 | 156.8 |
|  |  | PUFA ~ selection | 4 | 172.7 |
|  |  | PUFA ~ assay | 4 | 165.4 |
|  |  | PUFA~ 1 | 3 | 182.5 |

**Table S4.** Fatty acid contents (µg g^-1^ dry weight) of *Thalassiosira* sp*.*, *Chaetoceros tenuissimus*, *Synedra* sp. and *Chaetoceros* sp. under different treatments. S 26 ^o^C: long-term (2 years) ambient temperature (26 ^o^C) selected cells assayed at 26 ^o^C; S 26 ^o^C-A 30 ^o^C: long-term (2 years) ambient temperature (26 ^o^C) selected cells assayed at 30 ^o^C; S 30 ^o^C-A 26 ^o^C: long-term (2 years) warming temperature (30 ^o^C) selected cells assayed at 26 ^o^C; S 30 ^o^C: long-term (2 years) warming temperature (30 ^o^C) selected cells assayed at 30 ^o^C. Values are the average of three replicates and the standard deviation (SD, in parentheses). u.d.= under detection limit, i.e. <0.1%.

| Species | Treatment | | | |
| --- | --- | --- | --- | --- |
| *Thalassiosira* sp. | S 26 ^o^C | S 26 ^o^C-A 30 ^o^C | S 30 ^o^C-A 26 ^o^C | S 30 ^o^C |
| C14 | 2919.6 (282) | 4311.2 (800) | 5896.5 (1275) | 7288.2 (264) |
| C15 | 1031.6 (92) | 26.8 (2) | 1823.6 (437) | 1723.1 (81) |
| C15:1 n9 | u.d. | 1427.2 (146) | u.d. | u.d. |
| C16 | 14833.3 (1140) | 3118.7 (171) | 16984.1 (1296) | 15256.7 (1378) |
| C17 | u.d. | u.d. | u.d. | 442.1 (34) |
| C16:1 n7 | 9997.5 (1033) | u.d. | 15349.2 (1184) | 13495.4 (1338) |
| C16:2 n6 | 194.4 (47) | 401.3 (83) | 469.5 (120) | 198.7 (57) |
| C16:2 n4 | 457.6 (95) | 646.5 (127) | 1005.8 (247) | 506.7 (123) |
| C18 | 595.8 (76) | 122.0 (12) | 283.0 (58) | 943.2 (27) |
| C19:1 | 280.2 (37) | u.d. | 126.9 (23) | 323.6 (16) |
| C16:3 n3 | 968.1 (223) | 550.3 (60) | 2249.2 (552) | 1132.8 (465) |
| C16:4 n3 | 380.8 (99) | u.d. | 737.9 (156) | u.d. |
| C18:1 n7 | 22.6 (3) | 32.4 (3) | 83.4 (14) | 125.6 (18) |
| C18:1 n9 | 2705.7 (301) | u.d. | 1102.4 (272) | 2648.4 (241) |
| C18:2 n6 | 297.4 (21) | 225.7 (29) | 687.4 (175) | 791.2 (169) |
| C18:3 n6 | u.d. | u.d. | 403.8 (99) | 676.9 (277) |
| C18:3 n3 | 176.7 (35) | 82.7 (7) | 56.3 (14) | 385 (149) |
| C18:4 n3 | 329.7 (59) | 61.4 (2) | 1379.9 (237) | 1294.7 (815) |
| C20:4 n6 | 361.4 (78) | u.d. | 577.3 (148) | 243.3 (106) |
| C20:5 n3 | 962.3 (304) | 673.2 (48) | 2680.6 (769) | 932.9 (523) |
| C22:5 n6 | 129.9 (37) | 43.8 (5) | 529.9 (113) | 342.3 (182) |
| C22:6 n3 | 236.0 (125) | 116.1 (19) | 373.9 (100) | 275.2 (136) |
|  |  |  |  |  |
| SFA (%) | 52.5 (1.3) | 64.0 (0.8) | 47.3 (4.0) | 52.3 (3.7) |
| MUFA (%) | 35.3 (0.4) | 12.3 (0.2) | 31.6 (0.3) | 33.9 (0.8) |
| PUFA (%) | 12.2 (1.5) | 23.7 (0.6) | 21.1 (3.8) | 13.8 (4.4) |
| SFA (μg/g) | 19380.3 (1577) | 7578.7 (975) | 24987.2 (2240) | 25653.3 (1607) |
| MUFA (μg/g) | 13006.1 (1371) | 1459.6 (148) | 16661.9 (1473) | 16592.9 (1584) |
| PUFA (μg/g) | 4494.1 (1034) | 2800.9 (287) | 11151.6 (2644) | 6779.8 (2974) |
|  |  |  |  |  |
| *Chaetoceros tenuissimus* |  |  |  |  |
| C14 | 2912.6 (67) | 796.4 (149) | 3789.1 (93) | 3528.8 (705) |
| C15 | 274.1 (32) | 87.2 (4) | 574.0 (12) | 494.3 (256) |
| C14:1 n5 | 7.61 (1) | u.d. | 31.6 (6) | u.d. |
| C16 | 1071.9 (74) | 370.7 (27) | 1780.0 (44) | 1231.6 (205) |
| C16:1 n7 | 1753.5 (175) | 557.3 (65) | 2686.2 (212) | 1656.1 (298) |
| C16:2 n6 | 184.4 (31) | 54.3 (7) | 323.5 (33) | 155.1 (30) |
| C16:2 n4 | 152.7 (27) | 55.9 (5) | 238.2 (25) | 129.5 (24) |
| C18 | 199.2 (18) | 115.9 (9) | 143.3 (6) | 247.7 (44) |
| C16:3 n3 | 242.4 (54) | 142.5 (17) | 616.1 (73) | 200.8 (40) |
| C16:4 n3 | 87.7 (20) | u.d. | 226.5 (22) | u.d. |
| C18:1 n9 | 669.9 (62) | 170.5 (11) | 26.0 (5) | 489.4 (99) |
| C18:1 n7 | u.d. | u.d. | 226.6 (46) | 48.7 (7) |
| C18:2 n6 | u.d. | u.d. | 234.7 (45) | u.d. |
| C18:4 n3 | 28.8 (4) | u.d. | 45.2 (7) | 16.2 (4) |
| C20:4 n6 | u.d. | u.d. | 32.8 (6) | u.d. |
| C20:5 n3 | 166.7 (27) | 102.5 (6) | 624.3 (19) | 125.1 (21) |
|  |  |  |  |  |
| SFA (%) | 57.5 (2.1) | 55.8 (1.3) | 54.2 (1.5) | 66.1 (0.9) |
| MUFA (%) | 31.4 (0.8) | 29.7 (0.7) | 25.6 (1.5) | 26.4 (0.6) |
| PUFA (%) | 11.1 (1.3) | 14.5 (1.3) | 20.2 (0.5) | 7.5 (0.3) |
| SFA (μg/g) | 4457.9 (188) | 1366.6 (176) | 6286.3 (114) | 5502.3 (1181) |
| MUFA (μg/g) | 2431.1 (236) | 727.8 (69) | 2970.4 (265) | 2194.2 (404) |
| PUFA (μg/g) | 862.7 (154) | 355.2 (29) | 2341.2 (129) | 626.6 (117) |
|  |  |  |  |  |
| *Synedra* sp |  |  |  |  |
| C14 | 8652.9 (2046) | 4420.1 (467) | 7542.4 (2736) | 7381.8 (1913) |
| C15 | 518.8 (116) | 196.1 (5) | 319.2 (106) | 206.5 (26) |
| C16 | 10484.5 (4031) | 3357.9 (233) | 6935.4 (2429) | 4445.6 (648) |
| C16:1 n7 | 10552.1 (2145) | 3067.5 (543) | 6968.1 (2959) | 4762.9 (615) |
| C16:2 n6 | 387.4 (76) | 166.4 (38) | 184.0 (73) | 199.8 (31) |
| C16:2 n4 | 397.5 (90) | 196.8 (42) | 168.2 (64) | 151.1 (10) |
| C18 | 303.7 (72) | 129.3 (17) | 129.9 (31) | 137.3 (8) |
| C16:3 n3 | 133.3 (15) | 138.9 (38) | 59.4 (18) | 196.1 (19) |
| C18:1 n7 | 30.4 (3) | 110.1(19) | 9.9 (5) | 102.5 (5) |
| C18:1 n9 | 152.6 (25) | u.d. | 72.4 (33) | u.d. |
| C16:4 n3 | 273.0 (39) | u.d. | 237.0 (38) | u.d. |
| C18:3 n6 | 64.8 (15) | 16.3 (3) | 42.1 (17) | 26.9 (3) |
| C18:4 n3 | 44.6 (9) | 15.4 (4) | 58.4 (10) | 15.2 (2) |
| C20:4 n6 | 25.2 (5) | 12.2 (3) | 9.7 (3) | 20.9 (1) |
| C20:5 n3 | 727.3 (150) | 286.2 (41) | 540.4 (146) | 311.3 (71) |
| C22:5 n6 | 101.4 (24) | 50.5 (8) | 77.9 (17) | 75.8 (17) |
|  |  |  |  |  |
| SFA (%) | 60.7 (3.3) | 66.6 (2.1) | 63.9 (2.5) | 67.5 (1.8) |
| MUFA (%) | 32.7 (2.6) | 26.1 (1.8) | 30.2 (2.6) | 27.0 (1.6) |
| PUFA (%) | 6.6 (0.7) | 7.3 (0.5) | 5.9 (0.9) | 5.5 (0.6) |
| SFA (μg/g) | 19959.9 (6260) | 8103.4 (708) | 14926.8 (5298) | 12173.2 (2571) |
| MUFA (μg/g) | 10735.1 (2166) | 3177.6 (561) | 7050.4 (2996) | 4865.4 (613) |
| PUFA (μg/g) | 2154.5 (375) | 882.6 (160) | 1377.2 (372) | 996.9 (147) |
|  |  |  |  |  |
| *Chaetoceros* sp. |  |  |  |  |
| C14 | 1149.4 (124) | 1995.9 (125) | 2355.8 (197) | 1236.5 (175) |
| C15 | 41.2 (14) | 497.8 (37) | 291.7 (6) | 306.6 (29) |
| C16 | 1653.4 (270) | 3241.5 (601) | 3178.6 (45) | 1855.8 (122) |
| C16:1 n7 | 1277.7 (248) | 3067.1 (401) | 17.5 (2) | 606.5 (106) |
| C16:2 n6 | 18.7 (5) | 64.6 (15) | 72.3 (7) | 143.7 (88) |
| C16:2 n4 | 98.3 (33) | 200.5 (38) | 208.9 (18) | 75.5 (1) |
| C18 | 338.9 (45) | 768.6 (41) | 443.6 (8) | 1146.0 (123) |
| C19:1 | 92.2 (26) | 376.6 (52) | 124.7 (10) | 185.3 (13) |
| C16:3 n3 | 7.3 (1) | 228.6 (85) | 53.5 (1) | 24.8 (3) |
| C18:1 n7 | 29.1 (5) | 58.6 (10) | 59.0 (8) | 70.2 (8) |
| C18:1 n9 | 583.5 (90) | 1836.2 (346) | 1441.9 (43) | 874.3 (164) |
| C18:2 n6 | 100.9 (24) | 343.8 (40) | 350.4 (28) | 604.9 (121) |
| C18:4 n3 | u.d. | 34.7 (28) | 31.5 (1) | 289.7 (53) |
| C20 | u.d. | u.d. | u.d. | 53.9 (6) |
| C18:3 n6 | 18.9 (4) | 107.7 (9) | 59.5 (6) | 38.6 (6) |
| C18:3 n3 | u.d. | u.d. | 25.9 (3) | 325.8 (64) |
| C22 | 8.5 (2) | 49.4 (4) | 11.7 (1) | 23.3 (3) |
| C20:4 n6 | u.d. | 151.7 (27) | 36.4 (4) | 60.1 (4) |
| C20:5 n3 | 95.7 (20) | 324.7 (54) | 166.1 (3) | 128.6 (26) |
| C24 | 51.7 (7) | 52.4 (3) | u.d. | u.d. |
| C22:5 n6 | u.d. | u.d. | u.d. | 119.1 (17) |
|  |  |  |  |  |
| SFA (%) | 58.3 (3.5) | 49.3 (1.7) | 70.4 (0.2) | 56.6 (3.3) |
| MUFA (%) | 35.6 (2.7) | 39.8 (1.0) | 18.4 (0.0) | 21.3 (3.2) |
| PUFA (%) | 6.1 (0.8) | 10.9 (0.8) | 11.3 (0.3) | 22.1 (1.0) |
| SFA (μg/g) | 3243.2 (459) | 6605.7 (708) | 6281.3 (153) | 4622.2 (325) |
| MUFA (μg/g) | 1982.4 (360) | 5338.5 (799) | 1643.1 (43) | 1736.2 (267) |
| PUFA (μg/g) | 339.8 (80) | 1456.2 (227) | 1004.5 (50) | 1810.9 (180) |
